# Transcriptomic and Functional Validation Reveals PAQR3/P6-55 as Potential Therapeutic Targets in Colon Cancer

**DOI:** 10.3390/biology14070780

**Published:** 2025-06-27

**Authors:** Xue You, Yikuo Gai, Ziyun Wang, Yanqi Wang, Jingran Ye, Yujia Cao, Hengshuo Zhang, Ziyi Zhang, Ying Feng

**Affiliations:** 1 Lin He’s Academician Workstation of New Medicine and Clinical Translation, Jining Medical University, 133 Hehua Road, Jining 272067, China; 2 College of Clinical Medicine, Jining Medical University, Jining 272067, China; 18865702697@163.com (Y.G.); w0408122024@163.com (Y.W.); 15966698536@163.com (J.Y.); cyj1275973555@163.com (Y.C.); 13287280269@163.com (H.Z.); 3 College of Medical Imaging and Laboratory, Jining Medical University, Jining 272067, China; m13581059603@163.com (Z.W.); 19853855612@163.com (Z.Z.)

**Keywords:** PAQR3, P6-55, PI3K-AKT, colon cancer

## Abstract

Colon cancer is one of the leading malignant tumors worldwide, and the membrane protein PAQR3 has been identified as a tumor suppressor in multiple cancers. Notably, the peptide synthesized from 6 to 55 amino acids at the N-terminal of PAQR3 (P6-55) has been shown to effectively inhibit the growth of gastric cancer cells. This study aims to elucidate the mechanism of PAQR3 and explore its therapeutic potential in colon cancer. CCK8 cell viability assays, colony formation assays, and transwell migration assays were employed to systematically assess the inhibitory effects of PAQR3 on the proliferation and migration of colon cancer cells. Furthermore, we confirmed that P6-55 exhibits functional similarities to PAQR3, effectively inhibiting the growth of colon cancer in vitro and in vivo. RNA sequencing revealed that PAQR3 suppresses tumor growth via the PI3K-AKT signaling pathway, providing a strong theoretical foundation for therapeutic strategies targeting PAQR3/P6-55. In conclusion, our findings highlight the therapeutic potential of PAQR3/P6-55 as novel colon cancer inhibitors.

## Simple Summary

This study explores the roles and therapeutic potential of Progestin and adipoQ receptor 3 (PAQR3) in colon cancer. It was found that the peptide segment P6-55, synthesized from 6 to 55 amino acids at the N-terminus of PAQR3, functions similarly to PAQR3 and effectively inhibits the growth of colon cancer both in vitro and in vivo. RNA sequencing indicated that PAQR3 suppresses tumor growth via the PI3K-AKT signaling pathway, providing a theoretical basis for therapeutic strategies targeting PAQR3/P6-55.

## 1. Introduction

Colorectal cancer (CRC) is a common malignancy of the digestive tract, ranking among the top three cancers due to its high global incidence and mortality rates. Statistics show that approximately 10% of new cancer cases worldwide are attributed to CRC each year, leading to nearly 900,000 deaths annually, thus highlighting the serious threat that it poses to public health [1]. In recent years, its incidence has steadily increased, with a trend toward affecting younger populations. Existing treatment strategies primarily include surgical resection, chemotherapy, radiotherapy, targeted therapy, and immunotherapy [2]. While these approaches have extended survival and improved the quality of life for patients to some extent, the often subtle early symptoms of this disease result in many cases being diagnosed at advanced stages, contributing to a persistently low five-year survival rate [3]. Additionally, tumor heterogeneity and drug resistance significantly limit the efficacy of existing treatments. Given this serious situation, exploring new therapeutic targets and developing innovative treatments have become increasingly urgent.

PAQR3 (Progestin and adipoQ receptor 3)—also known as RKTG (Raf Kinase Trapping to Golgi)—is a key member of the PAQR family and widely recognized for its role as a tumor suppressor. As a membrane protein with a 7-transmembrane structure, it is localized to the Golgi apparatus, with its N-terminus facing the cytoplasm and C-terminus facing the Golgi lumen. PAQR3 plays a crucial tumor-suppressive role across various cancer types. Previous studies have revealed that PAQR3 interacts with Raf-1 and anchors it to the Golgi apparatus, thereby blocking transmission via the downstream MEK-ERK signaling pathway. Through this spatial segregation mechanism, PAQR3 effectively inhibits the sustained activation of the Ras-Raf-MEK-ERK pathway, a highly active pro-proliferative signaling cascade in most cancer cells [4]. Subsequently, mounting evidence has revealed that PAQR3 not only regulates the MAPK pathway but is also closely associated with multiple core cancer pathways, including PI3K-AKT, NF-κB, and TGF-β/Smad [5,6,7].

The expression of PAQR3 is commonly downregulated in various tumors and is significantly associated with poor patient prognosis. In gastric cancer, research has shown that PAQR3 facilitates the interaction between Twist1 and the ubiquitination ligase BTRC, accelerating Twist1 degradation and ultimately inhibiting epithelial–mesenchymal transition (EMT) and metastasis [8]. It is noteworthy that PAQR3 exhibits significantly low expression in gastric cancer tissues, and its expression is negatively correlated with tumor size, stage, metastasis, and patient survival rate [9]. In non-small cell lung cancer (NSCLC), PAQR3 suppresses tumor growth through multiple mechanisms. On the one hand, it enhances erlotinib-induced autophagy by blocking the interaction between BECN1 and activated epidermal growth factor receptor (EGFR), thereby inhibiting tumor cell growth and survival [10]. On the other hand, PAQR3 also induces apoptosis by regulating the NF-κB/p53/Bax signaling pathway, further suppressing tumor cell proliferation and survival [6]. Additionally, it can inhibit the PI3K/AKT signaling pathway, reducing tumor cell proliferation [5]. PAQR3 can also enhance ferroptosis by suppressing LDLR expression to inhibit the PI3K-AKT pathway, thereby restraining the progression of DLBCL [11]. PAQR3 can inhibit ALL proliferation and exacerbate ferroptosis by interacting with Nrf2 to modulate its stability [12]. Overall, PAQR3 demonstrates significant tumor-suppressive effects in multiple cancers, mediated by mechanisms including the inhibition of cell proliferation and EMT, regulation of autophagy, and induction of apoptosis. These mechanisms act synergistically to provide a solid scientific basis for PAQR3 as a potential therapeutic target, highlighting both the universality and specificity of PAQR3 as a tumor suppressor across different cancer types.

In recent years, the targeting relationship between the PI3K-AKT pathway and tumors has attracted widespread attention [13,14,15,16]. The PI3K-AKT axis serves as a crucial pro-survival pathway in most cancers, with its activation often being associated with p110α (PIK3CA) mutations, PTEN loss, or the hyperactivation of upstream receptor tyrosine kinases [17]. A study has revealed that PAQR3 competitively binds to the PI3K catalytic subunit P110α through its N-terminal region, thereby blocking its interaction with the regulatory subunit P85 and subsequently inhibiting PI3K kinase activity and reducing the phosphorylation level of downstream AKT. This mechanism has not only been validated in gastric cancer but also provides new insights for designing PI3K-targeted therapeutic molecules. Based on the discovery that the N-terminal region (amino acids 6-55) of PAQR3 binds to P110α and suppresses the growth of gastric cancer, a synthetic peptide (P6-55) mimicking this region was designed [18]. This peptide retains the critical binding domain with P110α and effectively emulates PAQR3’s PI3K-inhibitory function, significantly suppressing the proliferation of gastric cancer cells and tumor formation. However, it remains crucial to investigate whether PAQR3 and its synthetic peptide (P6-55) exhibit similar tumor-suppressive properties in CRC. A systematic investigation into the biological functions of PAQR3/P6-55 in CRC holds significant importance for expanding the therapeutic indications of these molecules, elucidating their mechanistic basis and facilitating their clinical translation.

We selected two colon cancer cell lines and successfully constructed stable cell lines with both PAQR3 overexpression and knockdown. Through a series of cell proliferation and migration assays, we confirmed that both PAQR3 and the P6-55 peptide exert significant tumor-suppressive effects in colon cancer cells. To further elucidate the tumor-suppressive mechanism of PAQR3, we conducted in vivo experiments, complemented by RNA sequencing and detailed data analysis. These studies not only reinforce our initial findings but also reveal that PAQR3 inhibits colon cancer growth by modulating the PI3K-AKT signaling pathway. This discovery provides new insights into the tumor-suppressive role of PAQR3 and highlights its potential as a therapeutic target for the development of precision treatments for colon cancer.

## 2. Material and Methods

### 2.1. Cell Culture, Lentiviral Packaging, and Stable Strain Construction

The cells used in the experiment were sourced from the Cell Bank of the Chinese Academy of Sciences. All cells were cultured in a 5% carbon dioxide and 37 °C constant-temperature incubator, with the regular culture medium being DMEM (C11995500BT, Gibco, Thermo Fisher Scientific Inc., Waltham, MA, USA), supplemented with 10% fetal bovine serum (UB68506-050, Bioodin, HanQiang (Canton) Biotechnology Co., Ltd., Guangzhou, China) and 1% dual antibiotics (penicillin/streptomycin). When the density of the HEK293T cells reached approximately 70%, the lentiviral packaging plasmids PSPAX2 and PMD2G were co-transfected with the core plasmid carrying the PAQR3 overexpression sequence or the shRNA for PAQR3 knockdown into HEK293T cells. PSPAX2 provided the Gag and Pol genes, which are essential for viral particle formation and maturation, while PMD2G supplied the VSV-G envelope protein to facilitate viral entry into host cells. Two core plasmids were employed: one carrying the PAQR3 overexpression sequence for overexpression, and the other containing shRNA targeting PAQR3 for knockdown. The cell culture supernatant was collected at 48 h and 72 h post-transfection, respectively. HCT116, HCT15, and SW480 cells were seeded into 6-well plates for culture. When the cell density reached approximately 40%, the collected viral supernatant was filtered using a 0.45 µm membrane (ISEQ00010, Millipore, Sigma-Aldrich, St. Louis, MO, USA). The filtered virus solution was then mixed with DMEM at a 1:1 ratio, and 10 µg/mL polybrene (H8761, Solarbio, Beijing, China) was added to infect the aforementioned colorectal cancer cells. At 48 h post-infection, puromycin was used to select the infected cells. After the cell density stabilized, qRT-PCR was performed to detect the overexpression and knockdown efficiency of PAQR3, thereby obtaining stable cell lines with either overexpression or knockdown of PAQR3.

### 2.2. CCK8 Experiment

Colon cancer cells were seeded into 96-well plates, with 1000 cells per well for HCT116 and 2000 cells per well for HCT15. Following the construction of stable strains or treatment with peptides at various concentrations, 10 µL of CCK8 reagent (GK10001, Glpbio, Montclair, CA, USA) was added to each well. Absorbance was measured at a wavelength of 450 nm using a microplate reader after incubation for 2 h.

### 2.3. Colony Formation Assay

For the colony formation experiments, 1000 or 500 cells were seeded into each well of either a six-well or twelve-well plate. Two weeks later, the cells were washed with PBS (WY1001, LANCOSA, Bela Rangpar, India), fixed with paraformaldehyde (BL539A, Biosharp, Tallin, Estonia) for 20 min, stained with crystal violet (C0121, Beyotime, Shanghai, China), and subsequently photographed and counted.

### 2.4. Cell Migration Assay

In the migration experiments using a transwell chamber (3422, Costar, Arlington, VA, USA), 3 × 10^5^ (HCT116) or 5 × 10^5^ (HCT15) cells were inoculated into the upper chamber containing 200 µL of serum-free DMEM after digestion and centrifugation. The lower chamber was filled with 600 µL of medium supplemented with 10% serum. After 24 h, the cells were washed with PBS, fixed with 4% paraformaldehyde for 20 min, and stained with crystal violet for an additional 20 min. The cells were then photographed and counted under a microscope.

### 2.5. RNA Extraction, Reverse Transcription PCR, and qRT-PCR

The colon cancer cells were lysed, and RNA was extracted using the Trizol (15596018, Ambion, Thermo Fisher Scientific Inc., Waltham, MA, USA) method. The extracted RNA was then reverse-transcribed into cDNA using a reverse transcription kit (RR047A, Takara, Kusatsu, Japan). The mRNA expression was quantified using a fluorescence quantitative PCR instrument, with GAPDH serving as a control. The primers used for fluorescence quantification are listed in Table 1.

### 2.6. Subcutaneous Tumor Formation Experiment in Nude Mice

The BALB/c-nude mice used in this study were obtained from Jinan Pengyue Experimental Animal Breeding Co., Ltd. (Jinan, China). All animal handling procedures were approved by the Animal Ethics Committee of Jining Medical University (Approval No. 2021-DW-ZR-008). Compliance with relevant guidelines and protocols was strictly maintained, and all procedures adhered to the ARRIVE guidelines. The peptides used in the experiment were synthesized by Synthbio Biotechnology Co., Ltd. (Hefei, China), and their sequences and usage methods were as previously described [18]. CRC cells were centrifuged, washed with PBS, and resuspended in serum-free medium. A total of 8 × 10^6^ cells were inoculated into the subcutaneous region on the right side of the nude mice. Once the tumor size reached 100 mm^3^, the mice were randomly divided into two groups. Control peptide and P6-55 were injected into the tumors every day, and the tumor volume was measured as previously described (Volume = length × width^2^/2) [18]. At the conclusion of the experiment, the tumors were excised and weighed prior to storage. The mice were sacrificed via carbon dioxide euthanasia, and the tumor tissue was finally collected.

### 2.7. Western Blot Analysis

Cells were lysed using RIPA lysis buffer (Beyotime, Shanghai, China) to extract proteins. The proteins were separated via SDS-PAGE gel electrophoresis. After activating the PVDF membrane, the proteins were transferred from the gel to the membrane. Following a blocking step, the primary antibodies were incubated overnight at 4 °C. The secondary antibody was then incubated at room temperature for 1 h. Finally, a developer was added, and detection was performed using the Amersham™ ImageQuant™ 800 (Cytiva, Marlborough, MA, USA). The antibodies used in the experiment are as follows: AKT (YT0185, Immunoway, Plano, TX, USA), p-AKT (YP0006, Immunoway), pI3K (YT6156, Immunoway), p-PI3K (YP0224, Immunoway), and GAPDH (10029187, Proteintech, Rosemont, IL, USA).

### 2.8. RNA Sequencing

Transcriptome sequencing was performed on RNA extracted using the Trizol method. Following concentration, purity, and integrity testing, the transcriptome sequencing library was constructed. Oligo(dT) magnetic beads were used to specifically capture mRNA, which served as a template for the sequential synthesis of first- and second-strand cDNA. The cDNA ends were then modified to create blunt ends. Subsequently, library fragments were ligated with NEBNext adapters (New England Biolabs, Ipswich, MA, USA) featuring hairpin structures and purified. After enzymatic reactions using USER Enzyme and PCR amplification, the PCR products were purified with AMPure XP magnetic beads (Beckman Coulter, Brea, CA, USA). The quality of the library was accurately evaluated using the Bioanalyzer 2100 system (Agilent Technologies, Santa Clara, CA, USA). Finally, high-throughput sequencing was conducted using the Illumina NovaSeq sequencing platform. Upon completion of sequencing, raw data were analyzed in-depth using a professional bioinformatics platform (www.biocloud.net). High-quality valid data were screened using Perl scripts, and these data were aligned to reference genome sequences using the Hisat2 (version 2.0.4) software. Differentially expressed genes were identified using the negative binomial distribution model in DESeq2, with the screening criteria set at a corrected *p* value of less than 0.05 and a fold change greater than or equal to 1.

### 2.9. GO Analysis, KEGG Pathway Enrichment Analysis, and GSEA

The clusterProfiler package, based on the Wallenius non-central hypergeometric distribution principle, was used to conduct GO enrichment analysis on the differentially expressed genes. KEGG pathway enrichment analysis was executed using the KOBAS database and clusterProfiler software (version 4.4.4) to assess the enrichment of differentially expressed genes within KEGG pathways. GSEA was conducted using OmicShare Tools (URL: https://www.omicshare.com/tools/Home/Soft/gsea, accessed on 24 June 2025) and involved sorting the transcriptome sequencing gene expression data from the control (*n* = 3) and PAQR3 knockdown (*n* = 3) HCT15 stable cell lines. By calculating the enrichment score (ES) and evaluating the significance of the differences, we revealed the biological processes that are affected by PAQR3 knockdown.

### 2.10. Statistical Analysis

All experimental data were analyzed using the GraphPad Prism 8.0 software. Comparisons between two groups were conducted using an unpaired two-tailed Student’s *t*-test. When comparing three or more groups, one-way analysis of variance (ANOVA) was performed, followed by Tukey’s HSD post hoc test to adjust for multiple comparisons and control the family-wise error rate. All data are presented as mean ± standard deviation (SD). All experiments were performed with a minimum of three biological replicates. *p* < 0.05 was considered statistically significant (*), *p* < 0.01 was deemed highly significant (**), and *p* < 0.001 was regarded as extremely significant (***).

## 3. Results

### 3.1. PAQR3 Knockdown Promotes the Proliferation and Migration of Colon Cancer Cells

**Figure 1:**
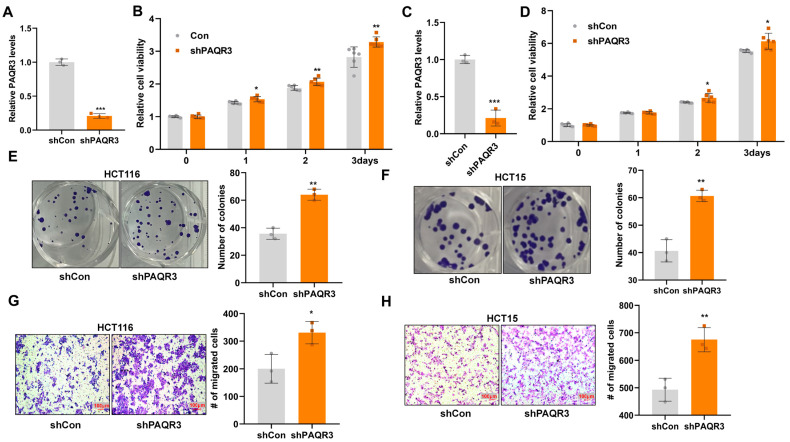
PAQR3 knockdown stimulates the proliferation and migration of colon cancer cells. (**A**) HCT116 cells were infected with lentivirus to achieve PAQR3 knockdown (PAQR3-KD), with a control group (shCon) for comparison. Following puromycin selection, the knockdown efficiency was confirmed via RNA extraction and subsequent qRT-PCR to measure mRNA expression levels. (**B**) The CCK8 assay was employed to assess the changes in the growth viability of PAQR3-KD and control HCT116 cells over the indicated time periods. (**C**) The knockdown efficiency of PAQR3 in HCT15 cells was similarly verified using qRT-PCR. (**D**) A CCK8 assay was conducted to further evaluate the growth viability of the two cell lines mentioned in (**C**). (**E**,**F**) Clonogenicity was assessed after inoculation of the PAQR3-KD and control HCT116 (**E**) and HCT15 (**F**) stable cell lines for 2 weeks. Representative images are displayed, with the number of clones quantified on the right. (**G**,**H**) A transwell chamber migration assay was performed to compare the migration capabilities of PAQR3 knockdown and control HCT116 (**G**) and HCT15 (**H**) cells. Representative images of crystal violet-stained cells are shown, with the statistical data for the number of migrating cells presented on the right. * indicates *p* < 0.05, ** indicates *p* < 0.01, and *** indicates *p* < 0.001.

To investigate the functional impact of PAQR3 knockdown in colon cancer cells, we successfully established stable PAQR3 knockdown (shPAQR3) and control (shCon) cell models (Figure 1A,C). CCK8 assays demonstrated that the downregulation of PAQR3 expression significantly enhanced the viability of both HCT116 (Figure 1B) and HCT15 (Figure 1D) cells. Furthermore, colony formation assays confirmed these results, indicating an increase in the number of colonies that were formed in both colon cancer cell lines following PAQR3 knockdown (Figure 1E,F). Additionally, migration assays revealed that the knockdown of PAQR3 also elevated the migratory potential of HCT116 (Figure 1G) and HCT15 (Figure 1H) cells.

### 3.2. PAQR3 Overexpression Inhibits the Proliferation and Migration of Colon Cancer Cells

**Figure 2:**
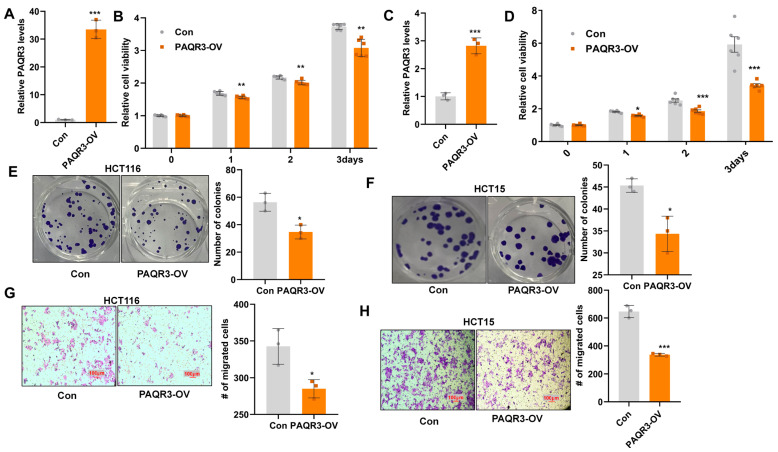
PAQR3 overexpression inhibits the proliferation and migration of colon cancer cells. (**A**) PAQR3 overexpression (PAQR3-OV) was achieved in HCT116 cells using lentiviral infection, with a corresponding control (Con) group being established. The efficiency of PAQR3 overexpression was evaluated via qRT-PCR. (**B**) After stable overexpression of PAQR3 in HCT116 cells, the stable cell line of A was seeded into 96-well plates, and the cell viability within 3 days was detected via CCK8 assay at a wavelength of 450 nm. (**C**) Stable cell lines with PAQR3 overexpression in HCT15 cells were constructed using the method described in A, and the expression efficiency of PAQR3 after stable overexpression in HCT15 cells was detected using qRT-PCR. (**D**) Changes in cell viability following PAQR3 overexpression in HCT15 cells were assessed at a wavelength of 450 nm through CCK8 assay. (**E**,**F**) The clonogenic ability of stably PAQR3-overexpressing HCT116 (**E**) and HCT15 (**F**) cell lines was assessed two weeks after inoculation. Representative images are displayed, with the number of clones quantified on the right. (**G**,**H**) The effect of PAQR3 overexpression on cell migration ability was determined using the transwell method. After stable overexpression of PAQR3 in HCT116 (**G**) and HCT15 (**H**) cells, cells were seeded into the upper chamber, and the cells that migrated to the lower chamber were fixed with paraformaldehyde for 20 min and stained with crystal violet for 20 min. After scrubbing, the number of cells migrating from the upper chamber to the lower chamber was determined under a microscope. Representative images are shown, along with statistical data for the number of migrating cells presented on the right. * indicates *p* < 0.05, ** indicates *p* < 0.01, and *** indicates *p* < 0.001.

To further investigate the functional properties of PAQR3, we explored its potential tumor-inhibitory effects by constructing PAQR3 overexpression (PAQR3-OV) and corresponding control (Con) models in colon cancer cells. Specifically, we selected two colon cancer cell lines—HCT116 and HCT15—and performed qRT-PCR to evaluate the efficiency of PAQR3 overexpression in these cells (Figure 2A,C). CCK8 assays subsequently confirmed the significant inhibitory effect of PAQR3 overexpression on the viability of colon cancer cells (Figure 2B,D). Moreover, our experimental results demonstrated that PAQR3 overexpression significantly reduced the colony-forming ability of these cancer cells (Figure 2E,F), highlighting its important role in inhibiting tumor growth. Additionally, we observed that the overexpression of PAQR3 effectively inhibited the migratory capability of colon cancer cells, further supporting its potential role in suppressing cancer progression (Figure 2G,H).

### 3.3. P6-55 Inhibits the Proliferation and Migration of Colon Cancer Cells

**Figure 3:**
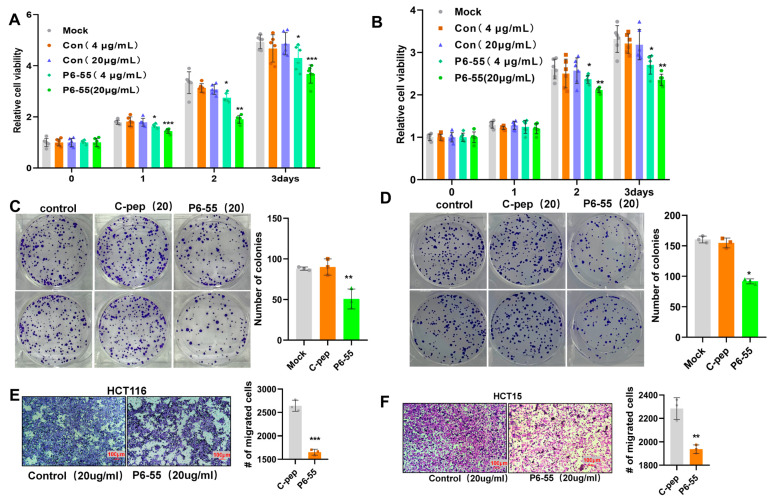
P6-55 inhibits the proliferation and migration of colon cancer cells. (**A**,**B**) HCT116 (**A**) and HCT15 (**B**) cells were treated with a control peptide or P6-55 for the indicated time periods, and cell viability was assessed using the CCK8 assay. (**C**,**D**) Colony formation was evaluated after treating HCT116 (**C**) and HCT15 (**D**) cells with a control peptide or P6-55 for 2 weeks, as demonstrated through crystal violet staining. Representative images are displayed, with the number of colonies quantified on the right. (**E**,**F**) The migration ability of the cells was tested using the transwell assay after treatment with a control peptide or P6-55. Representative images of migrating cells are shown, along with the statistical data for the number of migrating cells, presented on the right. * indicates *p* < 0.05, ** indicates *p* < 0.01, and *** indicates *p* < 0.001.

Given that P6-55 can substitute for PAQR3 to perform specific functions and effectively inhibit the growth of gastric cancer cells, we aimed to investigate whether P6-55 could similarly inhibit the proliferation and migration of colon cancer cells. The experimental results demonstrated that at a concentration of 4 μg/mL, P6-55 inhibited the proliferation of the colon cancer cell lines HCT116 and HCT15. Notably, increasing the concentration to 20 μg/mL resulted in a more pronounced inhibitory effect (Figure 3A,B). Furthermore, at a concentration of 20 μg/mL, the colony formation ability of the cells was significantly reduced (Figure 3C,D). Importantly, P6-55 also exhibited inhibitory effects on the migration ability of HCT116 and HCT15 cells (Figure 3E,F), reinforcing its potential role in combating the progression of colon cancer. Additionally, CCK8 and transwell assays conducted on another colon cancer cell line, SW480, revealed that P6-55 could significantly inhibit its proliferation and migration capabilities (Figure S1). This finding further consolidates the potential value of P6-55 in colon cancer treatment.

### 3.4. P6-55 Inhibits the Growth of Colon Cancer in a Nude Mouse Tumor Model

**Figure 4:**
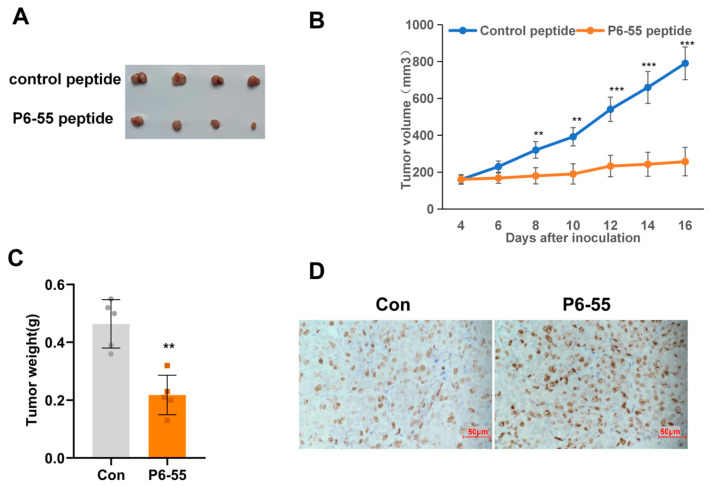
P6-55 inhibits the growth of colon cancer in a nude mouse tumor model. (**A**) HCT15 cells were injected subcutaneously into the right dorsal region of the mice in an equal number. When the tumor volume reached approximately 100 mm^3^, the mice were randomly divided into two groups. Both the control peptide and P6-55 were diluted with 50 μL of PBS at a dose of 100 mg, and each group received injections of either the control peptide or P6-55 every day. After 16 days of treatment, the tumors were excised and photographed. (**B**) The volume measurements of the tumors from mice treated with control peptide versus P6-55. (**C**) The weight measurements of the excised tumors from both treatment groups. (**D**) Immunohistochemical staining of Ki67 in paraffin-embedded sections of the tumor tissue shown in the subfigure (scale bar, 50 µm). ** indicates *p* < 0.01, and *** indicates *p* < 0.001.

To further confirm whether P6-55 inhibits tumor growth in vivo, we designed an experiment using subcutaneous tumor models to evaluate the impact of PAQR3 on tumor progression. Specifically, HCT15 cells were inoculated into the subcutaneous tissue of nude mice. Once the tumor volume reached approximately 100 mm^3^, the experimental mice were randomly divided into two groups, receiving treatments with either a control peptide or P6-55. The experimental results indicated that, compared with the control group, the P6-55 treatment significantly slowed the proliferation rate of HCT15 cells, effectively reducing both the volume and weight of the tumor (Figure 4A–C). Moreover, ki67 immunohistochemical staining of the tumor tissue also supported this conclusion (Figure 4D). Similarly, we once again validated the tumor-suppressive ability of P6-55 in vivo using another colon cancer cell line (Figure S2).

### 3.5. RNA-Seq and Differential Gene Enrichment Analysis

**Figure 5:**
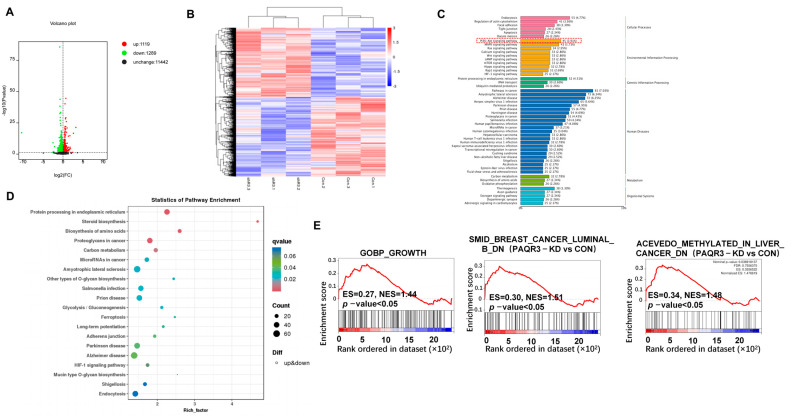
RNA-seq and differential gene enrichment analysis results. (**A**) A volcano plot illustrating the differential gene expression following RNA sequencing in HCT15 cells with stable PAQR3 knockdown compared with the control group. (**B**) A heat map comparing the expression profiles of differential genes between the PAQR3 knockdown and control groups. (**C**) KEGG classification diagram summarizing the pathways associated with differential genes that were impacted by PAQR3 knockdown. The red dashed frame indicates the enrichment of the PI3K-AKT signaling pathway. (**D**) A KEGG bubble plot highlighting the enrichment of differential genes that were affected by PAQR3 knockdown. (**E**) A GSEA plot demonstrating the biological functions that were influenced by PAQR3 knockdown.

To explore the tumor-suppressive mechanisms of PAQR3 in colon cancer, we performed RNA sequencing to compare the HCT15 stable cell line with PAQR3 knockdown with corresponding control cell lines. From the sequencing results following PAQR3 knockdown, a volcano plot was produced, which indicates that 1119 genes were upregulated, while 1289 genes were downregulated (Figure 5A). Furthermore, a heat map was produced, which visually represents the expression distribution of differential genes across the three control groups and the experimental groups, with red areas indicating high expression levels and blue areas indicating low expression levels (Figure 5B). We subsequently used the KEGG classification map to functionally annotate the differentially expressed genes and found that after PAQR3 knockdown, 45, 43, and 34 differential genes were significantly enriched in the key signaling pathways of PI3K-AKT, MAPK, and Ras, respectively. Additionally, 81 genes were closely associated with tumor-related pathways, while 38 genes were related to thermogenic pathways (Figure 5C and Figure S3). The KEGG bubble plot further illustrates that after PAQR3 knockdown, the differential genes were predominantly concentrated in processes such as protein processing, cholesterol biosynthesis, and amino acid metabolism (Figure 5D). Finally, GSEA indicated that after PAQR3 knockdown, growth- and tumor-related pathways exhibited an upregulation trend, providing new insights and a foundation for understanding the tumor-suppressive role of PAQR3 in colon cancer (Figure 5E).

### 3.6. PAQR3 Regulates the PI3K-AKT Signaling Pathway in Colon Cancer

**Figure 6:**
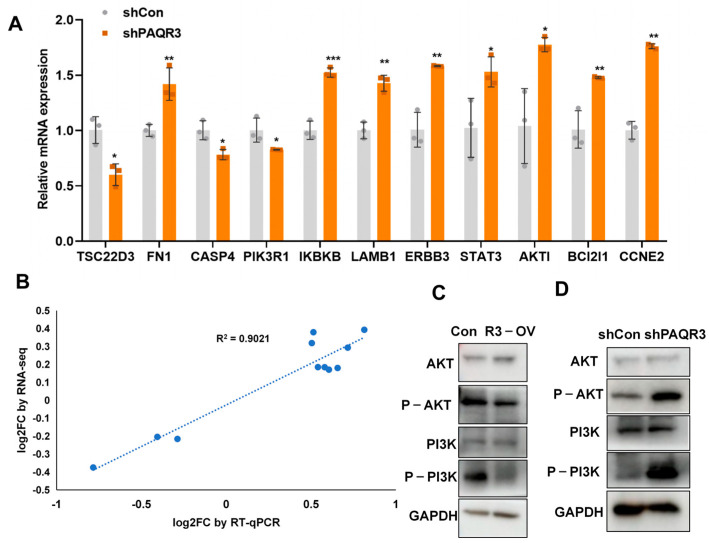
PAQR3 regulates the PI3K-AKT signaling pathway in colon cancer. (**A**) qRT-PCR analysis of the mRNA expression levels of differential genes associated with the PI3K-AKT signaling pathway in HCT15 cells with stable PAQR3 knockdown compared with the control group. (**B**) A correlation scatter plot comparing the results of RNA sequencing and qRT-PCR analyses. (**C**,**D**) Western blot (WB) analysis showing changes in protein expression levels following the overexpression (**C**) and knockdown (**D**) of PAQR3 in HCT15 cells, as depicted in the figure. The original images are shown in Figure S5. * indicates *p* < 0.05, ** indicates *p* < 0.01, and *** indicates *p* < 0.001.

Given that the KEGG classification diagram indicated significant enrichment of the PI3K-AKT signaling pathway following PAQR3 knockdown, we further explored whether PAQR3 knockdown regulates this pathway, thereby influencing tumor growth. To this end, we selected 11 genes that are closely associated with the PI3K-AKT signaling pathway for detailed analysis (Figure 6A). Comparing normal HCT15 stable cell lines and ones with PAQR3 knockdown using qRT-PCR, we found that the expression of AKT1, a core component of the PI3K-AKT pathway, along with the oncogenes IKBKB and STAT3, was significantly increased [19,20,21,22,23,24]. In contrast, the expression levels of the tumor suppressor PIK3R1 and the transcription regulator TSC22D3 were inhibited. Notably, TSC22D3 not only regulates cell growth, differentiation, and immune responses but may also partially inhibit anti-tumor immune responses [25]. PIK3R1, as a critical component of the PI3K-AKT pathway, is essential for cell growth, division, and other vital functions, and its expression also exhibited a downward trend following PAQR3 knockdown [26,27]. Additionally, we observed that the expressions of the cell adhesion-related genes FN1 and LAMB1 were upregulated after PAQR3 knockdown, aligning with the biological function of PAQR3 as a tumor suppressor gene [28,29,30]. The anti-apoptotic protein BCL2L1 and the pro-apoptotic CASP4, which play pivotal roles in cell survival and DNA damage response, showed expected changes, with increased BCL2L1 and decreased CASP4 levels being associated with PAQR3 knockdown [31,32,33]. The expression of the cell cycle-related gene CCNE2 was also increased, as anticipated [34,35]. Moreover, ERBB3—a receptor tyrosine kinase which is frequently overexpressed in various tumors and involved in cell proliferation and migration—demonstrated increased expression following PAQR3 knockdown [36]. In addition, we also established PAQR3-overexpressing and corresponding control cell lines in SW480 cells and found that PAQR3 significantly inhibited the PI3K-AKT signaling pathway (Figure S4).

Importantly, our quantitative results were highly consistent with the sequencing data (Figure 6B), further validating our findings. To confirm these results, we subsequently examined the inhibition and activation of the PI3K-AKT signaling pathway in HCT15 overexpression and knockdown stable cell lines, respectively (Figure 6C,D). The identification of the PI3K-AKT signaling pathway’s role in regulating the growth of colon cancer cells provides important insights for understanding the functional mechanisms of PAQR3 in colon cancer.

## 4. Discussion

Colon cancer is a prevalent malignant tumor of the gastrointestinal tract, with incidence rates rising annually. Currently, surgical resection remains the primary treatment; however, the high rates of recurrence and metastasis associated with this cancer present significant challenges. Therefore, elucidating the molecular mechanisms underlying the progression of CRC and identifying novel therapeutic targets are of paramount importance. By constructing stable cell line models of PAQR3 overexpression and knockdown in two colon cancer cell lines and then performing CCK8, colony formation, and transwell migration assays, we observed that PAQR3 significantly inhibits the proliferation and migration of colon cancer cells. Furthermore, we employed similar molecular and cellular biology techniques to demonstrate that the peptide P6-55 exhibits comparable effects on colon cancer cells. We further confirmed the inhibitory effects of PAQR3/P6-55 on the growth of colon cancer cells through RNA sequencing and functional validation. Notably, this study revealed PAQR3’s inhibitory effect on the PI3K-AKT signaling pathway in colon cancer cells, thereby elucidating its potential to inhibit tumor growth.

RNA sequencing analysis further revealed the complex signaling pathways that were affected by PAQR3 knockdown. In addition to the well-established PI3K-AKT signaling pathway, PAQR3 knockdown may significantly influence several other pathways, including MAPK, Ras, calcium, Wnt, cAMP, and mTOR. The abnormal activation of the Wnt signaling pathway in tumor cells is widely recognized as a key factor in promoting proliferation, metastasis, and epithelial–mesenchymal transition (EMT) [37,38]. Given the critical role and frequent abnormal activation of the PI3K-AKT-mTOR and MAPK/ERK signaling pathways in tumors, we speculate that PAQR3 may regulate the growth of colon cancer cells by inhibiting the PI3K-AKT-mTOR pathway, the MAPK/ERK pathway, or even the Wnt pathway [39]. Moreover, the sequencing results suggested that PAQR3 impacts the MAPK/ERK signaling pathway in CRC cells. Future studies could validate whether P6-55 does, indeed, influence this pathway and its interactions with Raf and Ras, as well as Raf and MEK. Additionally, the inhibitory effects of P6-55 could be assessed using a mouse model that spontaneously forms APCmin [40,41]. It is also worth investigating whether P6-55, as a PI3K inhibitor, exerts anti-tumor effects by targeting different signaling pathways.

Additionally, we observed that following PAQR3 knockdown, the expression of the anti-apoptotic protein BCL2L1 was upregulated, while the level of the pro-apoptotic protein CASP4 was reduced. This finding suggests that PAQR3 may influence the growth of colon cancer cells by regulating apoptotic processes [42]. Given the impact of PAQR3 knockdown on TSC22D3 (Figure 6A), we also observed that differentially expressed genes were enriched in the Herpes simplex virus 1 infection, Human T-cell leukemia virus 1 infection, Human immunodeficiency virus 1 infection, and Kaposi sarcoma-associated herpesvirus infection pathways (Figure 5C and Figure S4). Therefore, whether PAQR3 is associated with the tumor immune microenvironment remains a question to be explored in future studies. Previous studies have demonstrated that P6-55 initially garnered attention due to its significant inhibitory effects on the occurrence and progression of gastric cancer. Research employing real-time quantitative PCR technology to evaluate an anticancer fusion protein revealed its remarkable ability to regulate the expression levels of apoptosis-related genes in gastric cancer cell lines, effectively suppressing cancer cell proliferation and inducing apoptosis. These findings clearly demonstrate the application prospects of novel anticancer proteins in the field of tumor therapy [43]. Regarding P6-55, its potential impacts on tumor occurrence and development through different mechanisms warrant further investigation, with a focus on elucidating specific molecular pathways that are affected by this peptide. Considering that many chemotherapeutic agents have been associated with drug resistance during treatment, the ability of P6-55 to inhibit multiple signaling pathways that are critical to tumor progression highlights its potential as a valuable therapeutic agent. Furthermore, related studies have identified ASMTL-AS1 and LINC02604 lncRNAs as novel biomarkers for CRC, while SNHG7 also influences the progression of colorectal cancer by regulating the PI3K/Akt pathway [44,45]. Thus, exploring whether the use of P6-55 as a combinatory inhibitor alongside other chemotherapeutic drugs could provide enhanced therapeutic outcomes is another significant direction for future research [46,47,48,49]. From a translational perspective, further preclinical studies are necessary to evaluate the pharmacokinetics, bioavailability, and therapeutic efficacy of P6-55 in CRC models.

In conclusion, this study highlights the tumor-suppressive role of PAQR3 in CRC and reveals its regulatory impact on key oncogenic pathways. Our findings provide novel insights into the molecular mechanisms underlying the progression of CRC and suggest that targeting PAQR3 or utilizing its mimetic peptide, P6-55, may offer a promising avenue for the development of therapeutic strategies in future research.

## 5. Conclusions

This study demonstrated that the abnormal expression of PAQR3 triggers significant cancer progression, as evidenced by the results of the RNA-seq analysis and functional assays. We synthesized the PAQR3-derived peptide P6-55 and systematically validated its anti-tumor efficacy in both in vitro and in vivo models. Mechanistic investigations involving functional assays demonstrated that PAQR3 exerts anti-tumor effects by suppressing the PI3K-AKT signaling axis. This study not only demonstrated the PAQR3-PI3K/AKT regulatory axis through transcriptomic analysis with functional validation in colorectal cancer cells but also highlighted the potential of P6-55 as a candidate gene for the diagnosis and treatment of colorectal cancer.

Future research should focus on advancing the translational potential of P6-55 in colorectal cancer therapy through the following approaches:

Multi-omics profiling: Conduct proteomic and phosphoproteomic analyses to map PAQR3-regulated effectors and modulators, with a focus on its PI3K-AKT interaction hub, thus refining its tumor-suppressive network.
Pharmacological evaluation: Assess the pharmacokinetics, tumor-targeting efficiency, and pharmacodynamics of P6-55 in orthotopic and patient-derived xenograft models.
Evolutionary conservation: Use comparative genomics in non-mammalian models to identify the conserved therapeutic potential of PAQR3.
Prognostic analysis: Integrate genomic and proteomic datasets to evaluate the prognostic significance of PAQR3 in colorectal cancer cohorts.
Combination therapy: Test the synergistic effects of P6-55 with PI3K-AKT inhibitors or immune checkpoint blockers in immunocompetent models to develop precision treatments.

## Figures and Tables

**Table 1:** The primer sequences of the genes.

Genes	Forward Primer	Reverse Primer
*TSC22D3*	AACACCGAAATGTATCAGACCC	TGTCCAGCTTAACGGAAACCA
*FN1*	CGGTGGCTGTCAGTCAAAG	AAACCTCGGCTTCCTCCATAA
*CASP4*	CAAGAGAAGCAACGTATGGCA	AGGCAGATGGTCAAACTCTGTA
*PIK3R1*	AAGAAGTTGAACGAGTGGTTGG	GCCCTGTTTACTGCTCTCCC
*IKBKB*	GGAAGTACCTGAACCAGTTTGAG	GCAGGACGATGTTTTCTGGCT
*LAMB1*	AGGAACCCGAGTTCAGCTAC	CACGTCGAGGTCACCGAAAG
*ERBB3*	GACCCAGGTCTACGATGGGAA	GTGAGCTGAGTCAAGCGGAG
*STAT3*	CAGCAGCTTGACACACGGTA	AAACACCAAAGTGGCATGTGA
*AKT1*	AGCGACGTGGCTATTGTGAAG	GCCATCATTCTTGAGGAGGAAGT
*Bcl2l1*	GAGCTGGTGGTTGACTTTCTC	TCCATCTCCGATTCAGTCCCT
*CCNE2*	TCAAGACGAAGTAGCCGTTTAC	TGACATCCTGGGTAGTTTTCCTC

## Data Availability

The raw data from RNA-seq can be accessed through the NCBI’s SRA database, under accession number PRJNA1178261.

## Supplementary Materials

The following supporting information can be downloaded at: https://www.mdpi.com/article/10.3390/biology14070780/s1, Figure S1: P6-55 inhibits the proliferation and migration capabilities of SW480 cells. Figure S2: P6-55 can effectively inhibit the growth of subcutaneous tumors formed by HCT116 cells. Figure S3: A KEGG classification diagram showing the pathways associated with up- and downregulated differentially expressed genes after PAQR3 knockdown in HCT15 cells. Figure S4: The effect of PAQR3 on the PI3K-AKT signaling pathway in SW480 cells. Figure S5: Original Western blot figures for Figure 6.
