# Supplementary material for: Transcriptomic and Functional Validation Reveals PAQR3/P6-55 as Potential Therapeutic Targets in Colon Cancer
Source: Biology (Basel). 2025 Jun 27;14(7):780. doi: 10.3390/biology14070780 (PMC12292340; doi:10.3390/biology14070780)
Supplement: Supplementary file 1 [file biology-14-00780-s001.zip › biology-3649523-supplementary.pdf]

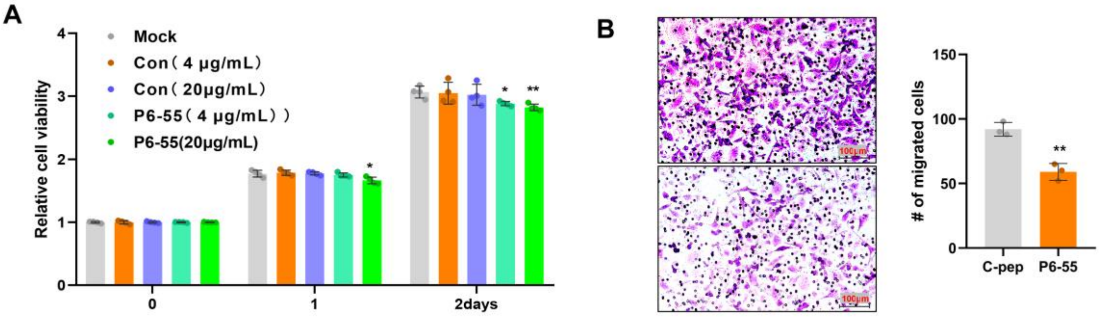

Figure S1. P6-55 inhibits the proliferation and migration capabilities of SW480 cells. (A) A total of 2000 SW480 cells were seeded in a 96-well plate and treated daily with peptides at the indicated concentrations. After adding CCK8 reagent for 2h, the OD values were measured at 450 nm absorbance. (B) SW480 cells were seeded in the upper chamber of a transwell apparatus with 200 µl serum-free medium, while the lower chamber contained 800µl serum-supplemented medium. After 24 hours of migration, cells remaining in the upper chamber were removed. The migrated cells were fixed with paraformaldehyde, stained with crystal violet, and quantified under a microscope.

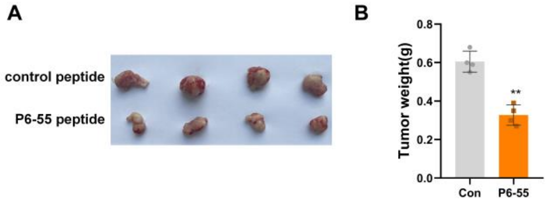

Figure S2. P6-55 can effectively inhibit the growth of subcutaneous tumors formed by HCT116 cells. (A-B) HCT116 cells were subcutaneously inoculated into the right flank of nude mice. When the tumor volume reached approximately 100 mm^3, the mice were randomly divided into two groups: one group received control peptide injections, and the other group received P6-55 peptide injections. After approximately 16 days of treatment, the tumors were harvested (A), and the tumors were weighed (B).

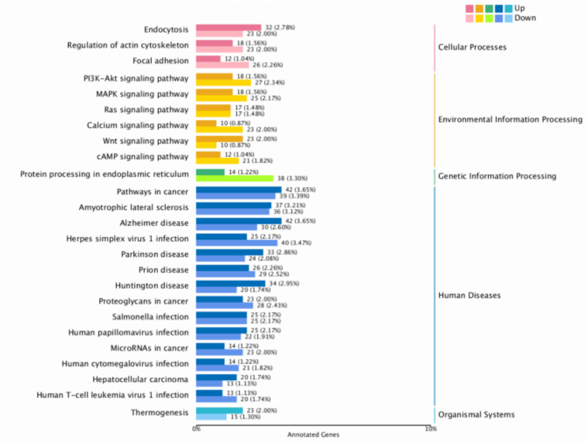

Figure S3. KEGG classification diagram showing the pathways associated with up- and downregulated differentially expressed genes after PAQR3 knockdown in HCT15 cells.

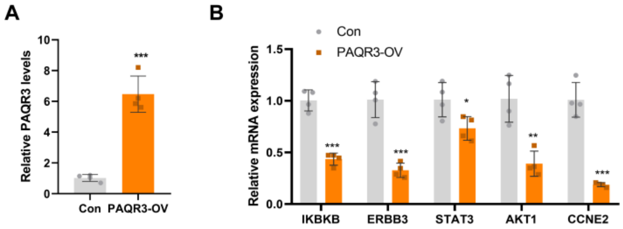

Figure S4. Effect of PAQR3 on the PI3K-AKT signaling pathway in SW480 cells. (A) SW480 cell lines with PAQR3 overexpression were established via lentiviral infection. (B) Cells from (A) were seeded in 12-well plates, and after RNA extraction and reverse transcription to cDNA, qRT-PCR was performed to detect the expression levels of the indicated genes.

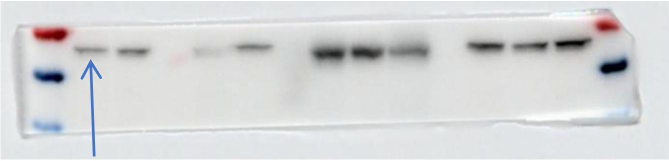

Figure 6C (AKT)

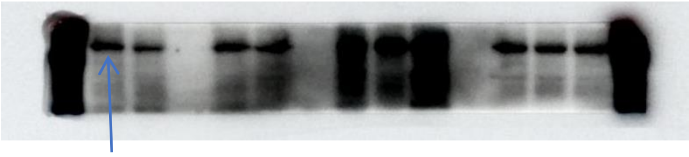

Figure 6C (P-AKT)

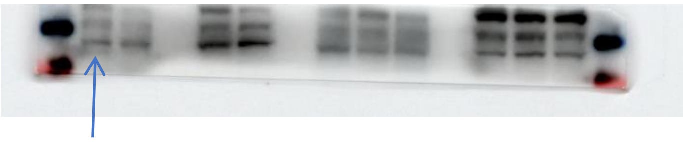

Figure 6C (PI3K)

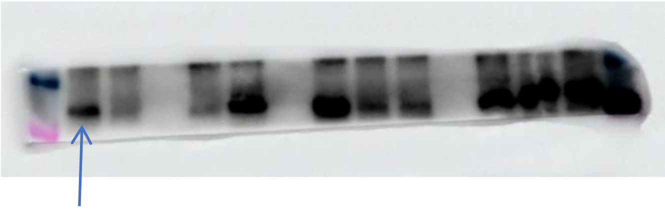

Figure 6C (P-PI3K)

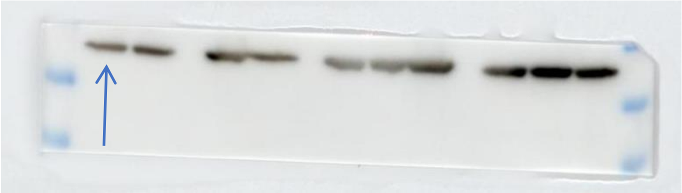

Figure 6C (GAPDH)

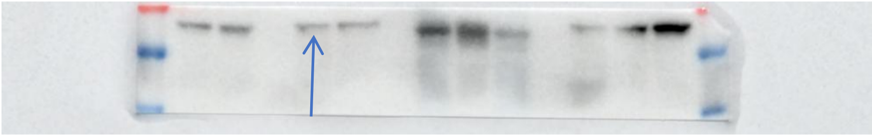

Figure 6D (AKT)

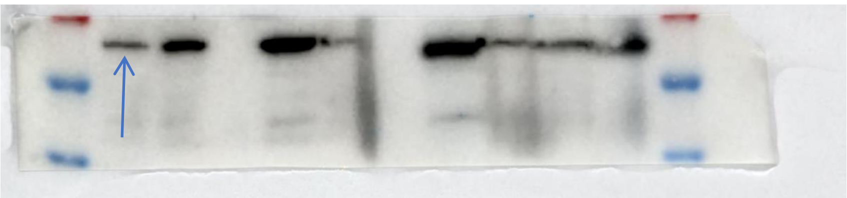

Figure 6D (P-AKT)

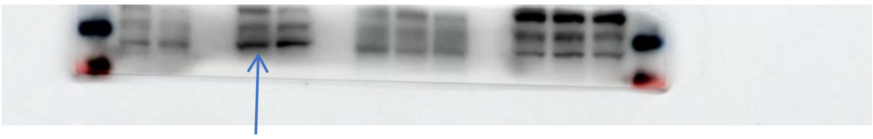

Figure 6D (PI3K)

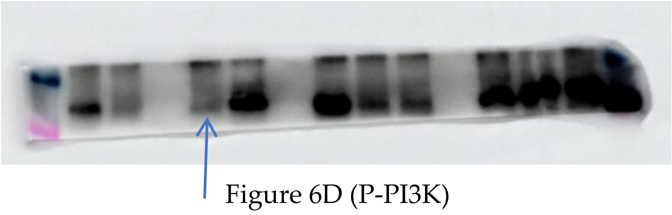

Figure 6D (P-PI3K)

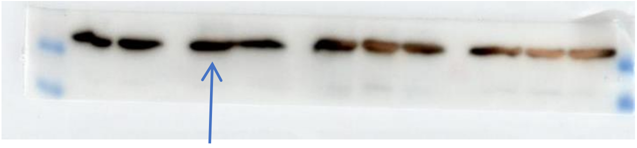

Figure 6D (GAPDH)

Figure S5. Original Western blot figures for Figure 6.
